# Geographic Distribution of *Epizootic haematopoietic necrosis virus* (EHNV) in Freshwater Fish in South Eastern Australia: Lost Opportunity for a Notifiable Pathogen to Expand Its Geographic Range

**DOI:** 10.3390/v11040315

**Published:** 2019-04-01

**Authors:** Joy A. Becker, Dean Gilligan, Martin Asmus, Alison Tweedie, Richard J. Whittington

**Affiliations:** 1 Sydney School of Veterinary Science, The University of Sydney, Camden 2570, Australia; joy.becker@sydney.edu.au (J.A.B.); alison.tweedie@sydney.edu.au (A.T.); 2 NSW Industry and Investment, Batemans Bay Fisheries Office, Batemans Bay 2536, Australia; dean.gilligan@dpi.nsw.gov.au; 3 NSW Industry and Investment, Narrandera Fisheries Centre, Narrandera 2700, Australia; martin.asmus@dpi.nsw.gov.au; 4 OIE Reference Laboratory for Epizootic Haematopoietic Necrosis Virus and Ranavirus Infection of Amphibians, Sydney School of Veterinary Science, The University Sydney, Camden 2570, Australia

**Keywords:** iridovirus, ranavirus, epidemiology, antibody, ELISA, virus isolation, prevalence, native-fish conservation, biosecurity, endemic disease

## Abstract

*Epizootic haematopoietic necrosis virus* (EHNV) was originally detected in Victoria, Australia in 1984. It spread rapidly over two decades with epidemic mortality events in wild redfin perch (*Perca fluviatilis*) and mild disease in farmed rainbow trout (*Oncorhynchus mykiss*) being documented across southeastern Australia in New South Wales (NSW), the Australian Capital Territory (ACT), Victoria, and South Australia. We conducted a survey for EHNV between July 2007 and June 2011. The disease occurred in juvenile redfin perch in ACT in December 2008, and in NSW in December 2009 and December 2010. Based on testing 3622 tissue and 492 blood samples collected from fish across southeastern Australia, it was concluded that EHNV was most likely absent from redfin perch outside the endemic area in the upper Murrumbidgee River catchment in the Murray–Darling Basin (MDB), and it was not detected in other fish species. The frequency of outbreaks in redfin perch has diminished over time, and there have been no reports since 2012. As the disease is notifiable and a range of fish species are known to be susceptible to EHNV, existing policies to reduce the likelihood of spreading out of the endemic area are justified.

## 1. Introduction

*Epizootic haematopoietic necrosis virus* (EHNV) is an aquatic pathogen that has been of international concern since its emergence in the early 1980s and is listed by the World Organization for Animal Health [1]. Infection with EHNV is systemic and causes epizootic hematopoietic necrosis (EHN), a disease characterized by extensive visceral tissue damage leading to mortality [2,3]. The first epidemics caused by EHNV occurred in freshwater impoundments in central Victoria (VIC), including Lake Mokoan and Nillahcootie in the Broken River catchment. Several populations of wild redfin perch (*Perca fluviatilis*) were impacted in the period from 1984 to 1986 [4,5]. These first epidemics often resulted in the mortality of tens of thousands of juvenile redfin perch and, in some outbreaks, adult fish were also affected. From 1986 to the mid-1990s, outbreaks of EHN in redfin perch and farmed rainbow trout (*Oncorhynchus mykiss*) were reported across the Murray–Darling Basin (MDB) in southeastern Australia [6].

Taxonomically, EHNV is in the genus *Ranavirus* within the Alphairidovirinae subfamily, Family Iridoviridae [7]. EHNV is only known in Australia, but similar viruses have been detected in other countries, usually associated with severe diseases in fish or amphibians [8]. EHNV is a double-stranded-DNA virus that can be released from a host cell by lysis (nonenveloped virion) or budding (enveloped virion) [9]. The genome of EHNV is 125 to 127 kb, with 106 to 109 genes, which is larger than amphibian ranaviruses that are usually 105 kb [7,10]. EHNV can be detected by virus isolation in several cell lines [4,11], antigen-capture ELISA [12], and histological sections using specific anti-EHNV antibodies [2]. Further, there are several polymerase chain-reaction (PCR) assays targeting the major capsid protein [13,14,15] or polymerase gene [16,17].

EHNV is extremely resistant and can survive for months or years in water or frozen fish [18,19]. It may persist in sediments and equipment for lengthy periods. Transmission of EHNV between susceptible hosts is possible via water or ingestion of tissue from infected fish [18,20,21]. EHNV infects fish through the skin, gills, or mouth. Infection results in multifocal necrosis of the hematopoietic tissue in the spleen, liver, and kidney [2,5,22,23]. Most infected fish quickly succumb and die within a few weeks, but disease expression is highly dependent on water temperature [21] and host [20,24]. Experimental challenge studies have demonstrated the potential for EHNV infection in several other species of Australian native fishes, including Murray cod (*Maccullochella peelii*), Macquarie perch (*Macquaria australasica*), and Murray River rainbowfish (*Melanotaenia fluviatilis*) [20]. In addition, freshwater fish native to Europe, such as the black bullhead (*Ameiurus melas)*, pike perch (*Sander lucioperca)*, and northern pike (*Esox lucius*) are susceptible to EHNV [25,26,27]. The combined results of Langdon [18] and Becker et al. [20] suggest that susceptibility of fish species does not appear to correlate with taxonomy, so susceptibility of untested species cannot be predicted based on taxonomic relatedness.

The MDB is considered to be one of the largest catchments in the world, with the river system flowing nearly 3750 kilometers from headwaters to the sea. The basin consists of 23 river valleys covering approximately 14% of Australia’s total land mass, with an area of just over one million square kilometers [28]. Mortality rates in initial EHNV epidemics in VIC and NSW were estimated to be >90% and, in some smaller water bodies, 100% mortality in redfin perch was reported [4,5,6]. From these outbreaks, there was evidence of both downstream and upstream spread of EHNV, as well as an instance outside of the MDB catchments [6]. Mechanisms of spread in redfin perch included fish migration, water movements, translocation of live redfin perch by recreational fishers, and mechanical vector transmission by piscivorous birds [19]. EHNV was distributed between water catchments in NSW with shipments of rainbow-trout fingerlings from two infected hatcheries in NSW in the 1990s [29], leading to biosecurity policy being implemented by the NSW government’s fisheries department to prevent this.

The occurrence of EHN has been discontinuous over time and space, with discrete disease events occurring within the endemic regions without a long-term pattern of recurrence. After spreading to new locations, the incidence of disease has been infrequent [10]. There have been no reports of EHNV since an outbreak in redfin perch in NSW since 2009, ACT since 2011, and VIC in 2012 [30].

Despite its significance, very little is known about the natural distribution of EHNV in Australia, or its potential impact on native fish, as no formal surveys have ever been conducted. Impacts of disease are very difficult, time-consuming, and costly to measure in natural aquatic systems, which has deterred research investment. The objective of this study was to investigate the distribution and make inferences about the epidemiology of EHNV in wild populations of fish species in the MDB. These comprise both introduced (alien) species, such as the redfin perch, and many native species.

## 2. Materials and Methods

### 2.1. Study Design

**Figure 1:**
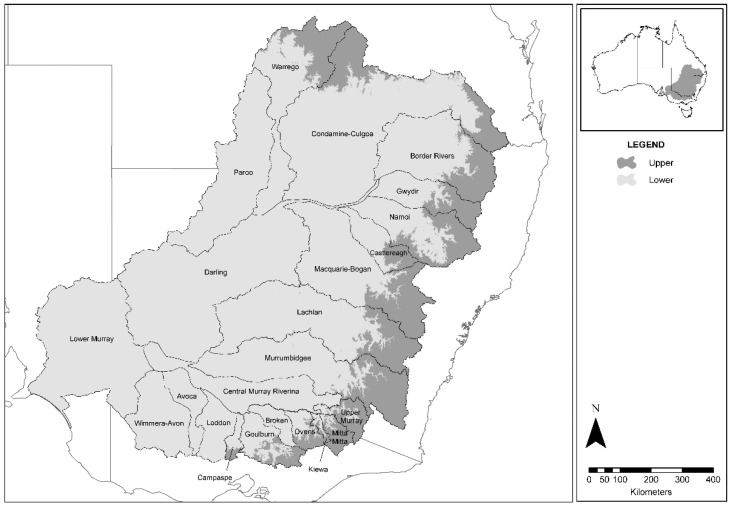
Maximum extent of study area and catchments of the Murray–Darling Basin.

Fish samples provided for laboratory testing were collected during established research programs by collaborating government agencies. In NSW, the Department of Primary Industries (NSW DPI) collected samples under the MDB Authority Sustainable Rivers Audit (SRA) (Figure 1) [31]. These samples were categorized according to fish species, collection year, river catchment, and altitudinal zone. In each zone, 7 fish sampling sites were chosen using a stratified random procedure, with a minimum of 18 sites per river catchment. Each was the centerpoint of a one-kilometer stream reach. This design was adopted following power and benefit–cost analyses of species-accretion data from the SRA [32]. Fish were sampled by boat-mounted or backpack electrofishing using standardized effort and methods. Sampled catchments in 2008 were Border Rivers, Broken, Darling, Loddon, Lower Murray, Mitta, Murray-Riverina, and Upper Murray. Sampled catchments in 2009 were Avoca, Goulburn, Kiewa, Lachlan, Macquarie-Bogan, Namoi, Paroo, and Warrego. Sampled catchments in 2010 were Campaspe, Castlereagh, Condamine-Culgoa, Gwydir, Murrumbidgee, Ovens, and Wimera-Avon. Other samples in NSW were collected from major impoundments that received an annual stocking of native fish and trout from several other independent research projects, and from disease investigations following notification of mortality events.

The Arthur Rylah Institute (https://www.ari.vic.gov.au/) conducted annual fish sampling across three catchments, representing three to four altitudinal zones. Australian Capital Territory (ACT) Municipal Services undertook sampling under its Urban Lakes Monitoring Program, as well as specific collections.

**Figure 2:**
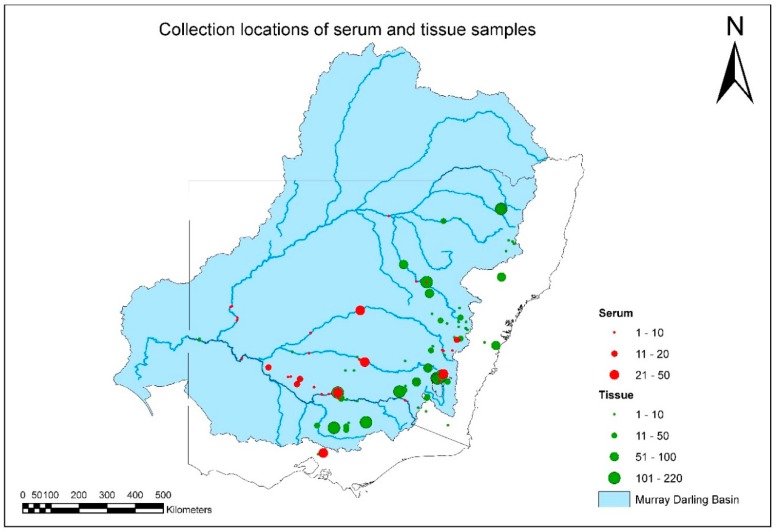
Distribution and number of tissue and serum samples collected from all fish species between July 2007 and June 2011.

Additional sites were sampled because of the presence of species that are potential hosts for EHNV, for example, Cataract Dam in the Nepean catchment, NSW (Macquarie perch); Hunter River, NSW (Australian bass), Snowy River, NSW (rainbow trout); Yarra River, VIC (redfin perch and Macquarie perch) (Figure 2 and Table 1). 

### 2.2. Collection of Fish Tissue and Serum

Fish were euthanized and were kept whole or dissected in the field, individually bagged (as practicable) and labeled with collector, date, species, and location details. These samples were held on ice or in portable refrigerators until return to a research station where some were dissected. Alternatively, whole fish were frozen at −20 °C until dissection at the University of Sydney (Camden, NSW), usually within six months of collection. For transport, frozen fish or tissue were packed with ice bricks and transported to Camden by road. During dissection, the spleen, posterior kidney, and liver were removed and placed into individual sample vials. All fish tissue was kept frozen at −20 °C until it was tested for EHNV.

For serum collection, fish were placed in plastic holding tubs and anesthetized with 20 mL of AQUI-S® per 100 L water until there was deep sedation evidenced by limited movement and complete loss of equilibrium. Blood was extracted from the caudal vein using an appropriate needle for the size of the fish (25–19 gauge), and was expelled into a 1.5 mL polypropylene tube (Eppendorf, Hamburg, Germany). Tubes were labeled and placed on ice or in a portable fridge until return to a research station. Whole blood samples were allowed to clot and were kept chilled for up to 7 days in the field. Samples of clotted blood were centrifuged at 2000 to 10,000 rpm for 10 min. The separated serum was removed to a labelled tube (Eppendorf) and placed at −20 °C. Serum samples were transported to Camden at −20 °C, thawed at room temperature, diluted 1:10 in 50% *v*/*v* glycerol in 25 mM Tris, 150 mM NaCl pH 7.4 with 0.02% *v*/*v* merthiolate (TSGM), and stored at −20 °C. Moribund or dead fish were sampled for virus isolation. Kidney, liver, and spleen were the target organs, pooled either after dissecting out the viscera, or by using the whole fish with the head and tail removed (for fish <40 mm total length).

### 2.3. Virus Isolation and Confirmation by PCR

Tissue samples were placed in sterile microcentrifuge tubes and stored at −80 °C if not processed immediately. Each sample was weighed, and 9 × weight/volume of homogenizing medium (HM) (minimum essential medium supplemented with 200 IU/mL penicillin, 200 µg/mL streptomycin, and 5 µg/mL Fungizone) was added. Tissue samples were prepared by grinding in a chilled mortar and pestle with sterile sand and HM, then clarified by centrifuging at 900× *g* for 10 min in a microcentrifuge. A 200 µL aliquot of the clarified homogenate was removed for DNA extraction, and a second 500 µL aliquot was prepared for virus isolation. It was further diluted 1:4 *v*/*v* in HM, passed through a 0.22 µm low protein-binding syringe-end filter, and used to inoculate bluegill fry (BF-2) cells in suspension in 24-well tissue culture plates. The cells were prepared by resuspending 80%–90% confluent cell monolayers in minimal essential medium supplemented with 10% fetal bovine serum (FBS), 200 IU/mL penicillin, 200 µg/mL streptomycin, and 5 µg/mL Fungizone to 2 × 10^5^ cells/mL. In duplicate, 150 µL of each sample was inoculated directly into a 1.5 mL cell suspension. Cells were incubated at 22 °C and examined for development of a cytopathic effect (CPE). If CPE developed, the infected tissue culture supernatant (TCSN) was harvested, 150 µL was passaged into a fresh cell suspension, and a 200 µL aliquot was reserved for DNA extraction to confirm the presence of EHNV by PCR. Any wells with cells that exhibited no CPE after 7–10 days were freeze-thawed at −20 °C overnight, and 150 µL of TCSN was passaged by well-to-well transfer into fresh cells. This was repeated after a further 7–10 day incubation to confirm samples as negative. DNA was extracted from 200 µL of TCSN using the HighPure Viral Nucleic Acid Extraction Kit (Roche, Basel, Switzerland), and examined using conventional or real-time PCR as described [1,13]. Results for individual samples were considered positive when either or both of the duplicates were positive.

### 2.4. Serology

Detection of specific anti-EHNV antibodies in fish serum was based on published methods [23]. All reagents were added to wells in volumes of 50 µL, and all incubations were at room temperature unless otherwise stated. A plain 96-well ELISA plate (Immulon®, Thermo Fisher Scientific, Waltham, MA, USA) was coated with affinity purified rabbit-anti-EHNV antibody in borate coating buffer, incubated for 90 min, then washed 5 times in reverse-osmosis purified water with 0.05% *v*/*v* Tween 20 using a plate washer (Wellwash 96-385, Thermo Electron Corporation, Waltham, MA, USA). Heat-inactivated EHNV antigen was added to pairs of wells in alternate pairs of columns on each plate to enable all sera to be tested in duplicate with and without antigen, and the plate was incubated overnight at 4 °C. After washing as above, the remaining binding sites were blocked in 1% *w*/*v* gelatin in phosphate-buffered saline pH 7.2 with 0.05% *v*/*v* Tween 20 (PBST) solution and incubated for 30 min. After washing, fish serum diluted in 0.01% *w*/*v* gelatin in PBST (PBSTG) was added and incubated for 90 min. Four positive-control sera from redfin perch were included; positive-control sera were not available for other species of fish. After washing, a sheep antifish immunoglobulin reagent appropriate for each species of fish, followed after washing by Donkey antisheep–horseradish peroxidase (HRP) conjugate (KPL) were added; both reagents were diluted in PBSTG, and incubations were for 90 min. After washing, the plate was developed with 1 mM 2,2’azino-bis(3-ethylbenzthiazoline-6-sulfonic acid) (ABTS) for 20 min before the reaction was stopped with 25 µL per well of 0.01% *w*/*v* NaN_3_ in 0.1 M citric acid. Optical density (OD) was read at 405 nm with a microplate reader (Multiskan Ascent, Thermo Electron Corporation). Signal-to-noise ratio (S/N) was determined as the mean OD for duplicate wells, with EHNV antigen divided by the mean OD for duplicate wells without EHNV antigen. A positive sample was defined when OD ≥ 0.4 and S/N ≥ 2 except in Macquarie perch, in which it was defined to be when OD > 0.6 and S/N ≥ 1.5.

### 2.5. Prevalence of EHNV Infection and Likelihood of Freedom of Infection

Populations were defined as groups of fish restricted by species, year of collection, river catchment, and zone (based on altitude as above or below 400 m) with collected fish from impoundments and lakes considered to be in distinct populations from those in rivers. The prevalence of EHNV infection in populations was calculated only where there were ≥30 samples. Maps were created using ArcMap® (ESRI, Redlands, CA, USA).

A fish was considered positive if a sample from it was positive in either virus isolation or ELISA. The prevalence of EHNV was defined as the proportion of fish samples that tested positive in that population and 95% exact binomial confidence intervals were determined using Minitab Statistical Software. The diagnostic sensitivity and specificity of virus isolation were both assumed to be 100% [6], so apparent prevalence equaled true prevalence. For ELISA, true prevalence and Blaker’s exact confidence limits were calculated assuming a test sensitivity of 70% and specificity of 100% using the calculator at http://www.ausvet.com.au.

For tissue samples tested by virus isolation where all test results were negative, the sample size required to be 95% certain that the population was free of EHNV at a specified design prevalence was determined using software at http://www.ausvet.com.au, specifically the modified hypergeometric exact calculation within the FreeCalc analysis results of freedom-testing function provided in the Survey Toolbox package [33]. Sample size was evaluated to obtain 95% confidence in detecting infection at a specified design prevalence. The following were specified as input parameters: Type I and II error levels, 0.05; sensitivity, 99.9%; specificity, 99.9%; population threshold for a binomial calculation, 10,000. For a population size of 10,000, the sample sizes required for design prevalence of 10%, 5%, 2%, and 1% were 29, 58, 145, and 270, respectively. For a population of 500, the sample sizes needed for the same design prevalence were 28, 55, 124, and 213. Where sample size was ≥30, these values were used to infer the prevalence that is not exceeded with 95% confidence at population sizes of 10,000 and 500.

For blood samples tested by ELISA, the same method to estimate sample size was used, but test sensitivity was assumed to be 70%. For a population of 10,000, sample sizes for 95% confidence at design prevalence of 10%, 5%, 2%, and 1% were 41, 84, 211, and 421, respectively. For a population of 500, sample sizes were 40, 78, 175, and 298.

## 3. Results

A total of 3622 fish-tissue samples were collected and tested over the duration of project (Figure 2, Table S1). EHNV was not isolated from any fish species other than redfin perch during this study (Tables S1 and S2). The geographic distribution and intensity of sampling is illustrated in Figure 2, and by species in Figures S1–S6. Overall MDB coverage was reasonable, but sample sizes in any year from each species and location were often small (Figure 2, Table 1).

For some populations of species such as the eastern mosquitofish (*Gambusia holbrooki*) in the upper Murrumbidgee, southern pygmy perch (*Nannoperca australisa*) in the upper Murray, and redfin perch in Lake Buffalo and the upper Gwydir (Copeton Dam), sample sizes were sufficient to suggest that EHNV is absent. That is, results showed that it is highly unlikely that, if present, EHNV exists in more than 1% to 2% of fish in each of these populations. For many other populations, this figure was <5% (Table 1).

**Figure 3:**
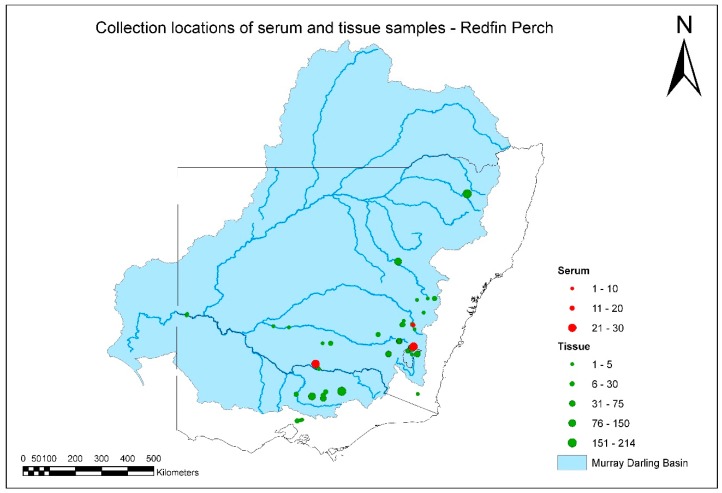
Distribution and number of tissue and serum samples collected from redfin perch between July 2007 and June 2011.

**Figure 4:**
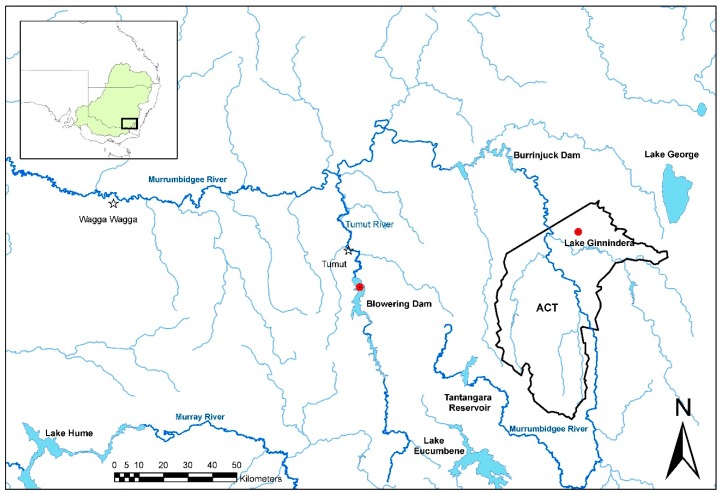
Moribund and dead redfin perch collected from Lake Ginnindera, ACT in December 2008 and 2010, and Blowering Dam, NSW in December 2009 were infected with EHNV.

A total of 1917 tissue and serum samples were collected from redfin perch during the project (Figure 3, Tables S1 and S2). There were several positive tissue samples from redfin perch, and all were from fish in the known endemic area in upper Murrumbidgee River catchment impoundments (Figure 4). One positive redfin perch was collected from Lake Ginninderra, ACT in December 2008 (Table 2). This sample was one of two dead redfin perch collected on 4 December 2008 near a boat ramp in Lake Ginnindera; the other dead fish and four that were electrofished at the site were negative for EHNV. It was unclear whether these fish were involved in an outbreak of EHN (Table 2). A further three positive redfin perch were from a group of 12, collected from Lake Ginninderra on 15 December 2010 (Table 2). Fish size was in the range of 30–63 mm total length and 0.3–2.6 g body weight (*n* = 12), and were the young of the year. In December 2009, eight positive redfin perch were identified from Blowering Dam, NSW collected during a fish kill. The estimated true prevalence was 7.3% (95% CI: 3.2%–14%; Table 1).

Longitudinally collected tissue samples from redfin perch were available from Lake Ginninderra (Table 2). EHNV infection was highly temporally clustered. In both 2008 and 2010, the virus emerged in the population between September/October and the end of December. Based on the sample sizes, the failure to detect EHNV in 44 and 36 adult redfin perch in October 2008 and February 2011, respectively, is consistent with <10% of individuals in those populations being infected at those times. Where EHNV was detected, the upper confidence limits for prevalence were 7.1% to 14.0% (Lake Ginninderra and Bowering Dam; Table 1).

A total of 492 blood samples were tested (Table S2). None of the blood samples from native fish was positive for anti-EHNV antibodies (Table S2; Figures S1–S3, S6). There were sufficient samples collected from Macquarie perch in the Yarra River, VIC to be 95% confident that, if present, EHNV infected <10% of fish in the population (Table 1; Figure S3). For Murray cod, there were sufficient samples collected to be 95% confident that EHNV prevalence was less than 5% in the lower Murray and <10% in the lower Lachlan rivers (Table 1 and Table S1).

A total of 142 serum samples, collected from redfin perch, were tested for anti-EHNV antibodies. The four positive control samples yielded positive results, with OD from 0.4 to >0.8, and S/N as high as about 6. There were two positive samples from the survey, both collected from adult redfin perch from Lake Ginninderra in February 2011 (Table S2). There were sufficient samples collected from redfin perch in the lower Murray river to be 95% confident that, if present, EHNV infected <10% of fish in the population (Table 1, Figure 3). As EHNV would be expected to infect a large proportion of redfin perch over time, this may be sufficient evidence to conclude that EHNV was absent from this population.

## 4. Discussion

Surveys to detect pathogens in wild fish in natural ecosystems are notoriously difficult, and methodological approaches are of international interest [34]. The main encountered problems include defining the population and obtaining representative samples. Consequently, it is easy to define the presence of a pathogen in a particular region when a disease is noticed, but very hard to be sure that a pathogen is absent when there are no signs of disease. A field survey to identify whether or not a pathogen is present in a population requires consideration of the likely prevalence of the pathogen if it is present (design prevalence), the sensitivity and specificity of diagnostic tests, the required degree of confidence in the results, and an ability to obtain representative samples, generally through random sampling. International standards for surveys to show that an infectious disease is not present have been proposed [35]. For diseases that are transmitted slowly or at an early stage of an outbreak, a design prevalence of 1% to 2% is recommended. For diseases that are highly transmissible, a design prevalence of 5% or more can be used. The present survey was conducted assuming that EHNV would be present in at least 10% of fish in a population. This was based on observations in redfin perch where the virus tends to affect a large proportion of the population [4,5]. It would be desirable to have much greater power of detection, down to 5%, 2%, or 1% prevalence, because, at those levels, claims about freedom from infection could be made with more certainty. An assumption was that the samples were random, but as this cannot be guaranteed, confidence limits may be wider than shown in Table 1. EHNV lacks host specificity so a range of fish species were targeted. However, it was logical to focus surveillance on the most susceptible known species [34], which is why the redfin perch was chosen to be the main target species in this survey.

Based on testing 3622 tissue and 492 blood samples, it was concluded that EHNV is endemic in some parts of the upper Murrumbidgee River catchment in the MDB. Other sites that were known to have infected redfin perch in the past were either not sampled (for example, Lake Hume, VIC) or tested negative (for example, Burrinjuck Dam, NSW, and Googong Reservoir, ACT). Although some samples were obtained from sites in Victoria near those that have previously harbored infected redfin perch (Broken River above and below Lake Nillahcootie in 2009), major potential sites of endemic infection were not sampled (for example, Lake Nillahcootie and Lake Mokoan). Similarly, lakes Albert and Alexandrina, and Mount Bold Reservoir in South Australia, all sites of past outbreaks [19], were not sampled. For these reasons, our current awareness of EHNV distribution in the known endemic region remains incomplete. However, it is reasonable to assume that it persists in places where it once occurred. This is because, during the study, an outbreak of disease due to EHNV occurred in juvenile redfin perch in Blowering Dam, NSW (December 2009), a dead redfin perch infected with EHNV was detected in Lake Ginninderra, ACT (December 2008), and there was an outbreak at this location in December 2010, consistent with prior observations of outbreaks in both locations [19]. There have been anecdotal reports of outbreaks in Blowering Dam and in the ACT since the last cases were formally confirmed in the 1990s. However, not all parts of the upper Murrumbidgee catchment were found to be infected in this study, for example, Cotter Reservoir was not. This is the location of an important population of endangered Macquarie perch, a species experimentally shown to be susceptible to EHNV [18].

The majority of samples from redfin perch came from the known EHNV-endemic area in the southeastern part of the MDB. It was not intended to obtain samples from South Australia or Queensland during this study, and fewer-than-expected samples were collected from redfin perch and any other species from northern catchments. This was partly because of the composition of the species assemblage sampled for the SRA. There are few populations of redfin perch in any of the northern catchments. Exceptions are the upper Gwydir catchment, from where a substantial number of samples of redfin perch were obtained, and a very small area of the upper Beardy River subcatchment of the Border Rivers catchment (Figure 3).

EHNV appeared to be absent from other species of fish in the MDB during the study period. Sufficient data were obtained from some regions of the MDB to be 95% confident that EHNV was present in <10% of individuals in population of the following species: river blackfish (*Gadopsis marmoratus*), brown trout (*Salmo trutta*), mountain galaxias (*Galaxias olidus*), eastern mosquitofish, murray cod, silver perch (*Bidyanus bidyanus*), southern pygmy perch, rainbow trout, and redfin perch.

The apparent absence of EHNV from most of the MDB and the demonstrated susceptibility of some fish species [18,20] should raise concern about the risk of its introduction. It is possible that the virus has not entered the middle and lower regions of southern MDB, nor its western and northern parts. However, the results of the survey only apply to the period of the survey, and sampling intensity was low for many species in most parts of the MDB.

Serology was applied in this survey, a procedure that is not routine in aquatic-animal health [36]. Two serum samples collected during this study were positive, as were many samples collected from redfin perch that survived experimental infection with EHNV, one of which was carrying the live virus in its organs [24]. The positive-control samples used in the assay had been collected from survivor redfin perch from Lake Mokoan in Victoria in 1991 to 1992, and were tested in 1991–1992 in prototype ELISA assays with similar results to those presented here (Whittington and Hyatt, unpublished). Therefore, the antibodies were stable in frozen storage (at −20 °C). These results indicate that serology does have application in field surveys for EHNV. Surveys for EHNV, where outbreaks of disease are intermittent even in endemic regions, reinforces the important role of antibody detection. Antibody detection provides historical evidence of exposure to the virus because antibodies persist in the blood of survivors long after the resolution of an active infection.

The life history of EHNV in nature is unclear. It is unknown whether fish can become infected with EHNV and remain carriers for life, and so is whether meaningful testing could be conducted when the disease is not apparent. It is also unclear whether an environmental reservoir of the virus may exist in river sediments, fish, or, indeed, in species other than fish. As the virus has been shown in laboratory experiments to be highly resistant to drying, and persists in water, on surfaces, and in frozen fish tissue, it has been assumed that it would also be highly resistant in the environment [18]. Whether environmental sources of EHNV are available to fish is completely unknown. It may, for example, persist in river sediments, but be tightly bound to particles and unavailable to fish. The results of examination of samples collected over time from redfin perch in Lake Ginninderra showed that EHNV could only be intermittently detected there. In two separate years, 2008 and 2010, the virus was undetectable in adult fish in September/October, but had appeared by the end of December in the young of the year. It is unknown whether the virus was truly absent from the adults, or whether it was present at a very low level, sufficient to initiate an outbreak in juvenile fish when conditions were appropriate for transmission. Larger sample sizes would be required to disprove a carrier state in adult redfin perch. Alternatively, there may be another reservoir host in Lake Ginninderra.

The spread mechanisms of EHNV in the natural environment are not entirely clear. However, it is certain that trade of infected rainbow trout fingerlings has been responsible for spread between farms in different catchments, for example, the upper Snowy, upper Murrumbidgee, and Shoalhaven Rivers [29], but it is unknown whether this has led to infection of wild fish. On the contrary, it is possible that farmed rainbow trout may become infected not only from infected fingerling trout, but also from wild redfin perch—this may explain annual outbreaks in rainbow trout farmed on the Tumut river adjacent to Blowering Dam [29]. Alternatively, the virus may persist on the farm in between detectable outbreaks. Downstream movement of EHNV seeming to not have historically occurred in the highly susceptible redfin perch population [19] or during this study tends to suggest that movement of the virus with water flow in rivers is unimportant, and that other factors are necessary for spread. These factors could include the anthropogenic movement of fish (live or dead redfin perch) or movement of the virus on equipment, by birds, or other putative vectors. An alternative view is that the disease caused by EHNV is naturally restricted to specific environments, predominantly lakes and impoundments. This is possible, and suggests the involvement of an amplification host or particular risk factors, such as locally high population density to assist transmission, particular temperature gradients, and unknown mechanisms.

The lack of evidence for the downstream spread of EHNV in rivers may be explained by dilution of the virus to a level below an infectious dose, insufficient host density in rivers to initiate propagation of an outbreak, or both, but the exact reasons are not known. Although density requirements and infectious dose are uncertain, it is reasonable to assume that a young redfin perch infected with EHNV could contain 10^7^ virions per gram; if the carcase is completely disaggregated in the water column, and the virus is not lost through adsorption to particulates, the concentration of virus in water in a 10 cm cube around a 10 g fish would be 10^4^/L, but would decrease rapidly as this water mixes with surrounding water. Assuming fish clustering within an impoundment and large water volume, it is clear that the virus becomes rapidly diluted. It is known that EHNV remains closely associated with fish tissue, in particular, the cell cytoskeleton [37], and so it may not be efficiently released. It may require, for example, ingestion of a fish carcase to transmit efficiently to another host. The natural occurrence of EHN in juvenile redfin perch while they are in dense schools is similar to the occurrence of EHN in rainbow trout in intensive culture. Such host density may be required for transmission between fish to occur, and may explain why outbreaks in native fish have not been seen. Native species generally do not occur in high density sympatric with redfin perch. Further research is required on this topic.

## 5. Conclusions

The conclusions from the study are that EHNV was probably confined to the upper Murrumbidgee River and associated impoundments in NSW and the ACT in 2007 to 2011, and these observations comprise the last reports of the virus from these areas. The possibility exists that EHNV is present in other areas of the MDB but was not detected, and in the locations in VIC where it originally emerged, although these were not sampled in this study. The last detection in Australia was in VIC in 2012. The reasons for the apparent disappearance of the virus are unknown, but policies to reduce the risk of exposure to EHNV of fish in the MDB are justifiable. It is logical to prevent or reduce the likelihood of spread from the endemic area to areas that are apparently free. Not all parts of the upper Murrumbidgee catchment, for example, Cotter Reservoir, are infected, and it would be desirable to maintain this freedom to protect the resident Macquarie perch population there. All reports of fish kills in the upper Murrumbidgee catchment region should be investigated and laboratory tests for EHNV undertaken. Routine surveillance of redfin perch populations in the upper Murrumbidgee catchment should be undertaken to determine the occurrence of EHNV. Policies to exclude recreational use of these areas during EHNV outbreaks, close fisheries, or enable compulsory disinfection should be considered.

## Figures and Tables

**Table 1:** Estimated true prevalence of *Epizootic haematopoietic necrosis virus* (EHNV) in redfin perch and other species based on testing tissue and serum samples collected between July 2007 and June 2011. Where sample size was >30 per population, and prevalence, which results indicate, was not exceeded for population sizes of 10,000 and 500. A population was defined as a group of fish restricted by species, year of collection, catchment, zone, and impoundment.

Population					Total No. of Samples	No. Positive	Prevalence %			Prevalence Level Not Exceeded with ≥95% Confidence Population of 10,000	Prevalence Level Not Exceeded with ≥95% Confidence Population of 500
Species	Collection Year	Compartment	Zone	Site Restriction			Point Estimate	95% Lower Confidence Limit	95% Upper Confidence Limit		
Redfin perch	2009	Broken			74	0	0.0	0.0	4.9	5	5
Redfin perch	2009	Goulburn			112	0	0.0	0.0	3.2	5	5
Redfin perch	2010	Gwydir	Upper	Copeton dam	153	0	0.0	0.0	2.4	2	2
Redfin perch	2008	Ovens (Murray)	Lower	Lake Buffalo	214	0	0.0	0.0	1.7	2	1
Redfin perch	2010	Macquarie	Lower		115	0	0.0	0.0	3.2	5	5
Redfin perch	2008	Murray	Lower		113	0	0.0	0.0	3.2	5	5
Redfin perch	2008	Murray	Lower		42^1^	0	0.0	0.0	10.8	10	10
Redfin perch	2009	Murray	Lower		84	0	0.0	0.0	4.3	5	5
Redfin perch	2009	Murrumbidgee	Upper	Blowering Dam	109	8	7.34	3.2	14.0	na	na
Redfin perch	2007	Murrumbidgee	Upper	Burrinjuck Dam	136	0	0.0	0.0	2.7	5	2
Redfin perch	2008	Murrumbidgee	Upper	Burrinjuck Dam	97	0	0.0	0.0	3.7	5	5
Redfin perch	2009	Murrumbidgee	Upper	Burrinjuck Dam	35	0	0.0	0.0	10.0	10	10
Redfin perch	2009	Murrumbidgee	Upper	Googong Reservoir	43	0	0	0.0	8.2	5	5
Redfin perch	2008	Murrumbidgee	Upper	Lake Ginnindera	51	1	1.96	0.0	10.4	na	na
Redfin perch	2009	Murrumbidgee	Upper	Lake Ginnindera	40	0	0	0.0	8.8	5	5
Redfin perch	2010	Murrumbidgee	Upper	Lake Ginnindera	120	3	2.50	0.0	7.1	na	na
Redfin perch	2011	Murrumbidgee	Upper	Lake Ginnindera	36	0	0.0	0.0	9.7	10	10
Redfin perch	2011	Murrumbidgee	Upper	Yerrabi pond	91	0	0.0	0.0	4.0	5	5
											
Australian bass	2009	Hunter^2^	Upper		98	0	0.0	0.0	3.7	5	5
Blackfish	2008	Murray	Lower		108	0	0.0	0.0	3.4	5	5
Brown trout	2009	Snowy^2^	Upper		34	0	0.0	0.0	10.3	10	10
Golden perch	2009	Gwydir	Upper	Copeton dam	80	0	0.0	0.0	4.5	5	5
Macquarie perch	2009	Yarra^2^			48^1^	0	0.0	0.0	9.5	10	10
Mountain galaxias	2009	Macquarie	Upper		48	0	0.0	0.0	7.4	10	10
Mountain galaxias	2008	Murrumbidgee	Upper		60	0	0.0	0.0	6.0	5	5
Mountain galaxias	2009	Lachlan	Upper		37	0	0.0	0.0	9.5	10	10
Mountain galaxias	2008	Murray	Upper		31	0	0.0	0.0	11.2	10	10
Eastern mosquitofish	2008	Murrumbidgee	Upper		419	0	0.0	0.0	0.9	1	1
Murray cod	2007	Lachlan	Lower		40^1^	0	0.0	0.0	11.4	>10	10
Murray cod	2008	Murray	Lower		102^1^	0	0.0	0.0	5.0	5	5
Murray cod	2008	Murrumbidgee	Upper	Blowering dam	31	0	0.0	0.0	11.2	10	10
Murray cod	2009	Murrumbidgee	Upper	Burrinjuck Dam	30	0	0.0	0.0	11.6	10	10
Rainbow trout	2008	Murrumbidgee	Upper		115	0	0.0	0.0	3.2	5	5
Rainbow trout	2009	Snowy^2^	Upper		31	0	0.0	0.0	11.2	10	10
Silver perch	2007	Murrumbidgee	Lower		32^1^	0	0.0	0.0	14.2	>10	>10
Silver perch	2009	Gwydir	Upper	Copeton dam	51	0	0.0	0.0	7.0	5	5
Southern pygmy perch	2009	Murray	Upper		229	0	0.0	0.0	1.6	2	1

na, not applicable; ^1^ serum tested by ELISA, remaining tests were by virus isolation; ^2^ not in the Murray–Darling Basin.

**Table 2:** Summary of results of virus-isolation tests for EHNV conducted on redfin perch tissue collected from Lake Ginninderra, ACT.

Date	No. of Fish	Total Length (mm)	EHNV
15 October 2008	44	190–480	not detected
5 November 2008	1	242	not detected
4 December 2008	6	43–57	1 infected
18 March 2010	7	100–114	not detected
17 September 2010	42	92–135	not detected
15 December 2010^1^	12	30–63	3 infected
15 December 2010^2^	35	49–189	not detected
19 December 2010	2	168–172	not detected
21 December 2010	2	125–157	not detected
21 February 2011	36	90–167	not detected

^1^ juvenile young of the year; ^2^ adult fish.

## Supplementary Materials

Supplementary materials can be found at https://www.mdpi.com/1999-4915/11/4/315/s1.
