# Supplementary material for: Geographic Distribution of Epizootic haematopoietic necrosis virus (EHNV) in Freshwater Fish in South Eastern Australia: Lost Opportunity for a Notifiable Pathogen to Expand Its Geographic Range
Source: Viruses. 2019 Apr 1;11(4):315. doi: 10.3390/v11040315 (PMC6520861; doi:10.3390/v11040315)
Supplement: Supplementary file 1 [file viruses-11-00315-s001.pdf]

Supplementary Table S1. Summary of tissue samples collected from redfin perch and other species between 2007 and 2011, based on year and catchment area.

Species	Collection Year	Compartment	Zone[1]	Site Locations	Number of Fish	
					Tested	VI Positive
Redfin perch	2007	Murrumbidgee	Upper	Burrinjuck Dam	136	
Redfin perch	2008	Goulburn	Lower	Tahbilk Lagoon	26	
Redfin perch	2008	Lachlan	Upper	Reidsdale, Little Narraway	5	
Redfin perch	2008	Lachlan	Upper	Boorowa River Site 860	25	
Redfin perch	2008	Ovens (Murray)	Lower	Lake Buffalo, VIC	214	
Redfin perch	2008	Murray	Lower	Mulwala Canal	113	
Redfin perch	2008	Murrumbidgee	Upper	Cotter River and Murrumbidgee River ACT	23	
Redfin perch	2008	Murrumbidgee	Upper	Burrinjuck Dam	97	
Redfin perch	2008	Murrumbidgee	Upper	Lake Ginnindera, ACT	51	1
Redfin perch	2008	Murrumbidgee	Lower	Columbo Creek	6	
Redfin perch	2008	Unknown			11	
Redfin perch	2009	Broken			74	
Redfin perch	2009	Gwydir	Upper	Copeton Dam	10	
Redfin perch	2009	Goulburn		Seven's Creek	112	
Redfin perch	2009	Lachlan	Upper	Boorowa River, Belubula River, Reidsdale, Narrawa Bridge, Abercrombie River	28	
Redfin perch	2009	Macquarie	Upper	Fish River, Campbells River	12	
Redfin perch	2009	Murray	Lower	Neds Anabranch, Yarrawonga to Tocumwal, Lake Victoria, Mulwala Canal	84	
Redfin perch	2009	Murrumbidgee	Upper	Cotter Dam	2	

Species	Collection Year	Compartment	Zone[1]	Site Locations	Number of Fish	
					Tested	VI Positive
Redfin perch	2009	Murrumbidgee	Upper	Cotter River	1	
Redfin perch	2009	Murrumbidgee	Upper	Molonglo River	7	
Redfin perch	2009	Murrumbidgee	Upper	Yerrabi Pond	2	
Redfin perch	2009	Murrumbidgee	Upper	Point Hut Crossing	1	
Redfin perch	2009	Murrumbidgee	Upper	Googong Reservoir	43	
Redfin perch	2009	Murrumbidgee	Upper	Lake Ginnindera	40	
Redfin perch	2009	Murrumbidgee	Upper	Blowering Dam	109	8
Redfin perch	2009	Murrumbidgee	Upper	Burrinjuck Dam	35	
Redfin perch	2009	Murrumbidgee	Lower	Bethungra Dam	6	
Redfin perch	2009	Murrumbidgee	Lower	Columbo Creek, Hay, Maude	9	
Redfin perch	2009	Snowy	Upper	Bombala	2	
Redfin perch	2009	Yarra[2]		Wonga Park, Jumping Creek	12	
Redfin perch	2010	Gwydir	Upper	Copeton Dam	153	
Redfin perch	2010	Macquarie	Lower	Dubbo	115	
Redfin perch	2010	Murrumbidgee	Upper	Burrinjuck Dam	16	
Redfin perch	2010	Murrumbidgee	Upper	Lake Ginninderra	120	3
Redfin perch	2011	Murrumbidgee	Upper	Yerrabi Pond	91	
Redfin perch	2011	Murrumbidgee	Upper	Lake Ginninderra	36	
Australian bass	2009	Hunter[2]	Upper	Glenbawn Dam	98	
River blackfish	2008	Murray	Lower	Mulwala Canal	108	
River blackfish	2008	Murrumbidgee	Lower	Tarcutta Creek	9	
Brown trout	2009	Macquarie	Upper	Lake Oberon	7	

Species	Collection Year	Compartment	Zone[1]	Site Locations	Number of Fish	
					Tested	VI Positive
Brown trout	2009	Snowy[2]	Upper	Swamp/Hughes Creek, Thredbo River (near Gaden Hatchery)	34	
Common carp	2009	Murray	Lower	Yarrawonga to Tocumwal	1	
Flat-headed gudgeon	2009	Murray	Lower	Mulwala Canal	28	
Flat-headed gudgeon	2009	Murrumbidgee	Upper	Blowering Dam	1	
Climbing galaxias	2008	Murray	Upper	Geehi River	1	
Mountain galaxias	2008	Murray	Upper	Geehi River	5	
Mountain galaxias	2008	Murray	Upper	Upper Mannus Creek	31	
Mountain galaxias	2008	Murrumbidgee	Upper	Pierces Creek, ACT	60	
Mountain galaxias	2009	Macquarie	Upper	Winburndale Rivulet	48	
Eastern mosquitofish	2008	Murrumbidgee	Upper	Cotter River, ACT	419	
Eastern mosquitofish	2011	Murrumbidgee	Upper	Yerrabi Pond	1	
Golden perch	2008	Murrumbidgee	Upper	Blowering Dam	20	
Golden perch	2008	Murrumbidgee	Upper	Burrinjuck Dam	22	
Golden perch	2008	Murrumbidgee	Upper	ACT	7	
Golden perch	2009	Murrumbidgee	Upper	Burrinjuck Dam	26	
Golden perch	2009	Gwydir	Upper	Copeton Dam	80	
Golden perch	2009	Murray	Lower	Yarrawonga to Tocumwal	3	
Golden perch	2009	Unknown		Victoria	2	
Golden perch	2010	Gwydir	Upper	Copeton Dam	8	
Macquarie perch	2009	Nepean[2]	Upper	Cataract Dam	27	
Mountain galaxias	2009	Lachlan	Upper	Retreat River	27	

Species	Collection Year	Compartment	Zone[1]	Site Locations	Number of Fish	
					Tested	VI Positive
Mountain galaxias	2009	Lachlan	Upper	Flyers Creek	37	
Murray cod	2007	Murrumbidgee	Upper	Burrinjuck Dam	10	
Murray cod	2007	Murrumbidgee	Lower	Buckingbong	3	
Murray cod	2008	Murray	Lower	Mulwala Canal	11	
Murray cod	2008	Murrumbidgee	Upper	Blowering Dam	31	
Murray cod	2008	Murrumbidgee	Upper	Burrinjuck Dam	13	
Murray cod	2009	Murrumbidgee	Upper	Burrinjuck Dam	30	
Murray cod	2009	Murray	Lower	Yarrawonga to Tocumwal, Lake Mulwala to Ovens River, Lake Hume to Lake Mulwala	29	
Murray cod	2009	Murrumbidgee	Lower	Biagee	1	
Murray cod	2009	Namoi	Lower	East Yarral, Miloo	3	
Murray cod	Unknown	Lachlan			1	
Murray cod	Unknown	Unknown			12	
Murray cod	2009	Gwydir	Upper	Copeton Dam	16	
Murray cod	2010	Murray	Lower	Yarrawonga	7	
Murray cod	2010	Murrumbidgee	Lower	Narranderra	13	
Oriental weatherloach	2009	Hawkesbury[2]	Lower	Little River	7	
Southern pygmy perch	2009	Murray	Upper	Coppabella Creek	229	
Rainbow trout	2008	Murrumbidgee	Upper	Cotter River and Condor Creek, ACT	115	
Rainbow trout	2009	Lachlan	Upper	Retreat River, Boree Creek	5	
Rainbow trout	2009	Macquarie	Upper	Lake Oberon, Turon River, Campbells River, Duckmaloi River	22	

Species	Collection Year	Compartment	Zone[1]	Site Locations	Number of Fish	
					Tested	VI Positive
Rainbow trout	2009	Snowy[2]	Upper	Gang Gang Creek	31	
Rainbow trout	2009	Namoi	Upper	McDonald River	4	
Roach	2009	Yarra[2]		Wonga Park	1	
Silver perch	2007	Murrumbidgee	Upper	Burrinjuck Dam	4	
Silver perch	2009	Gwydir	Upper	Copeton Dam	4	
Silver perch	2009	Murray	Lower	Yarrawonga to Tocumwal	7	
Silver perch	2009	Gwydir	Upper	Copeton Dam	51	
Silver perch	2010	Gwydir	Upper	Copeton Dam	4	
Trout cod	2009	Murray	Lower	Yarrawonga to Tocumwal	20	
Trout cod	2008	Murrumbidgee	Lower	Buckingboing boat ramp	1	
**TOTAL**					**3622**	**12**

[1]Upper (>400 m) and lower (<400 m) zones were designated based on altitude from Lintermans (2007); [2]not in Murray-Darling Basin.

Supplementary Table S2. Summary of serum samples collected from redfin perch and other species between 2007 and 2011, based on year and catchment area.

Species	Collection Year	Compartment	Zone[1]	Site Location	Number
Redfin perch	2008	Murray	Lower	Mulwala Canal	42
Redfin perch	2008	Lachlan	Upper	Reidsdale, Little Narraway	5
Redfin perch	2010	Murrumbidgee	Upper	Lake Ginninderra	8
Redfin perch	2011	Murrumbidgee	Upper	Lake Ginninderra*	14
Redfin perch	2011	Murrumbidgee	Upper	Yerrabi Pond	21
Golden perch	2008	Murrumbidgee	Upper	ACT	4
Golden perch	2010	Murrumbidgee	Upper	Lake Ginninderra	3
Golden perch	2011	Murrumbidgee	Upper	Lake Ginninderra	2
Golden perch	2011	Murrumbidgee	Upper	Yerrabi Pond	1
Macquarie perch	2008	Lachlan	Upper	Abercrombie River, Lachlan River	29
Macquarie perch	2009	Yarra[2]		Wonga Park, Jumping Creek	48
Murray cod	2007	Lachlan	Lower	Euabalong	40
Murray cod	2008	Murray	Lower	Colligen Creek., Edwards River, Murray River, Wakool River, Niemur River, Mulwala Canal	102
Murray cod	2008	Murray	Upper	Clarkes Reserve	3
Murray cod	2008	Darling	Lower	Minda, Menin Court, Cuthero Point, Whurlie	17
Murray cod	2008	Murrumbidgee	Lower	Hay boat ramp, Markey's Beach, Buckingbong	7
Murray cod	2008	Lachlan	Lower	Booligal Weir	4
Murray cod	2008	Macquarie	Lower	Dubbo boat ramp, Fred Firth boat ramp	4
Murray cod	2009	Namoi	Lower		7
Murray cod	2009	Namoi	Upper		5
Murray cod	2011	Murrumbidgee	Upper	Lake Ginninderra	10

Murray cod	2011	Murrumbidgee	Upper	Yerrabi Pond	10
Silver perch	2007	Murrumbidgee	Lower	NFC (Buckingbong)	32
Silver perch	2008	Murray	Lower	Colligen Creek, Edwards River, Murray River, Wakool River, Niemur River	23
Trout cod	2008	Murrumbidgee	Upper	ACT	4
				**TOTAL**	**445**

[1]Upper (>400 m) and lower (<400 m) zones were designated based on altitude from Lintermans (2007); * two samples were positive for antibodies specific for EHNV; [2]not in Murray-Darling Basin.

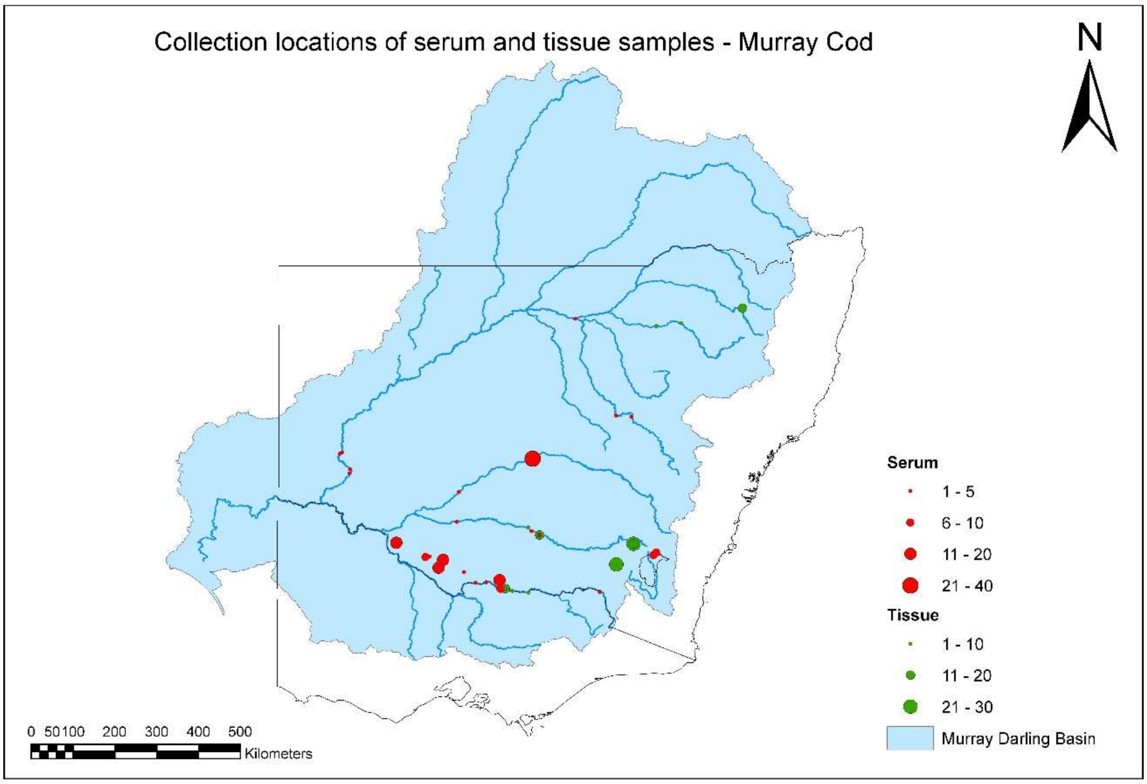

Supplementary Figure S1. Distribution and number of tissue and serum samples collected from Murray cod between July 2007 and June 2011.

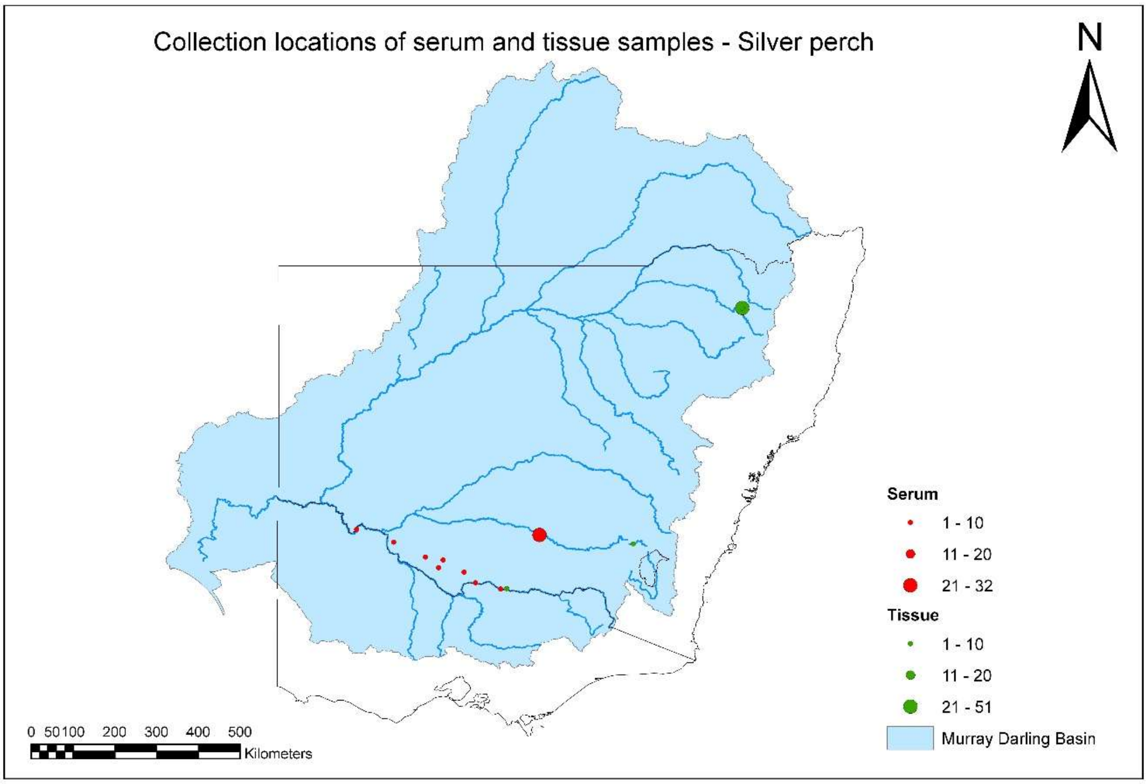

Supplementary Figure S2. Distribution and number of tissue and serum samples collected from silver perch between July 2007 and June 2011.

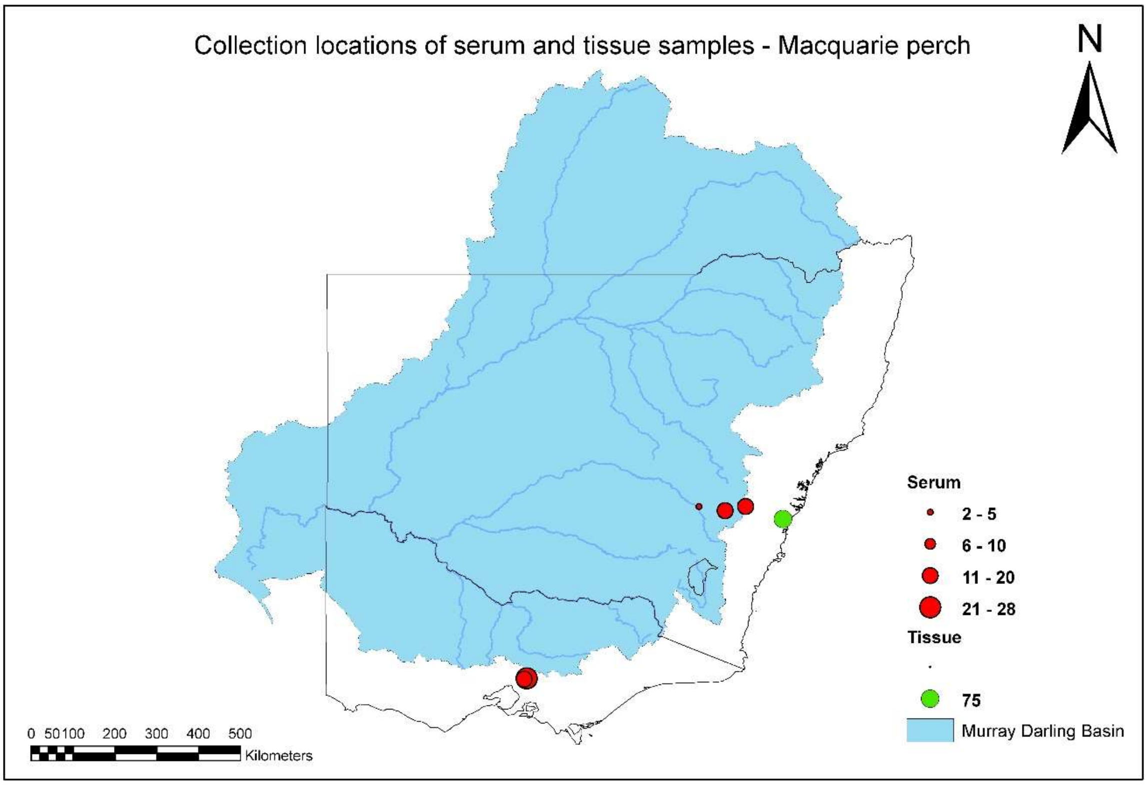

Supplementary Figure S3. Distribution and number of tissue and serum samples collected from Macquarie perch between July 2007 and June 2011.

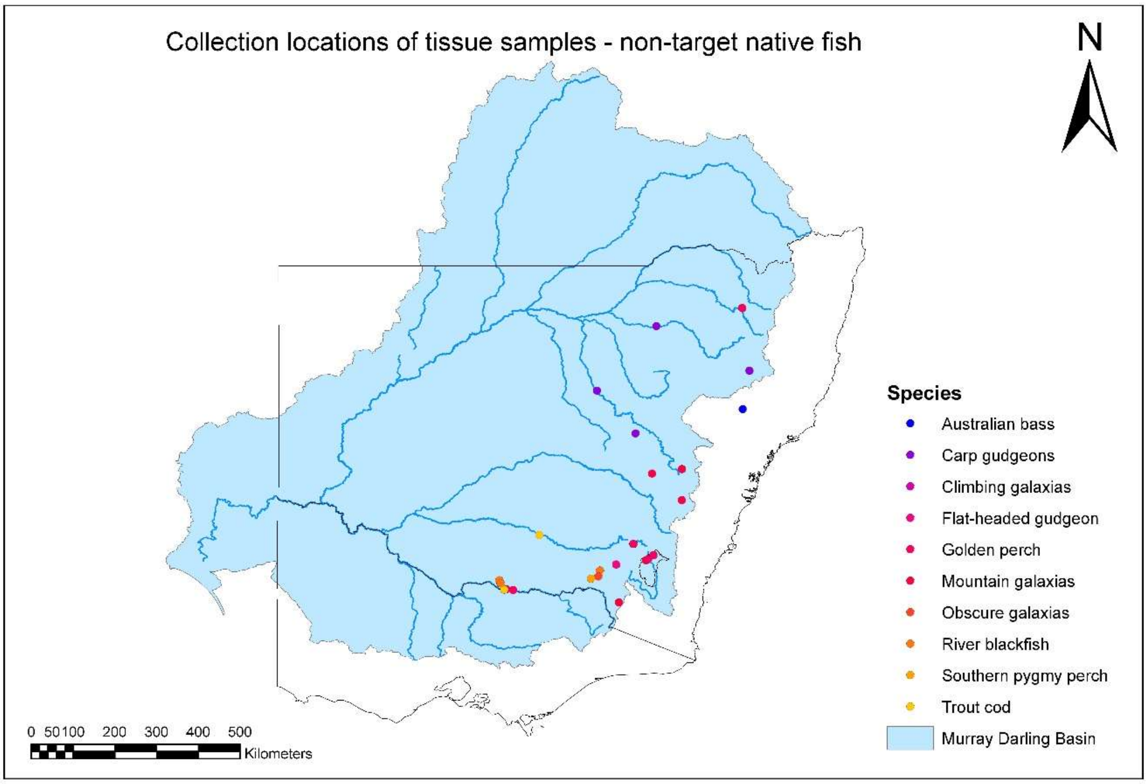

Supplementary Figure S4. Distribution of tissue and serum samples collected from various native fish species between July 2007 and June 2011.

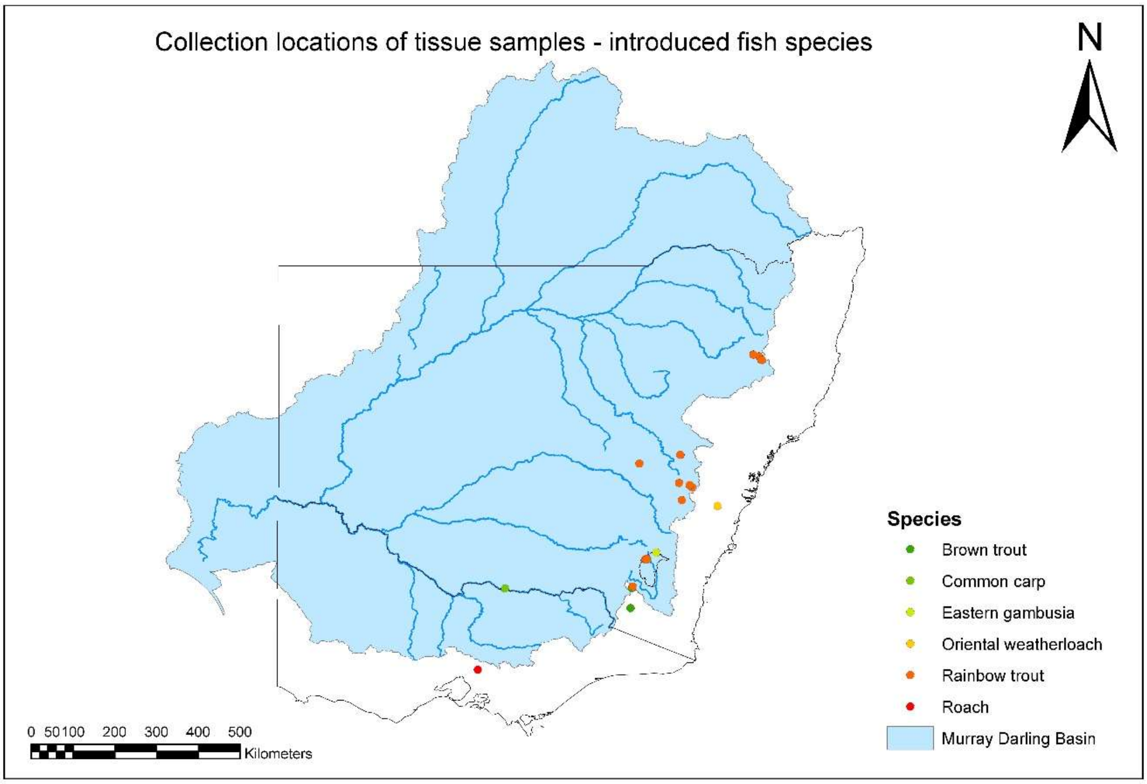

Supplementary Figure S5. Distribution of tissue and serum samples collected from alien fish species between July 2007 and June 2011.

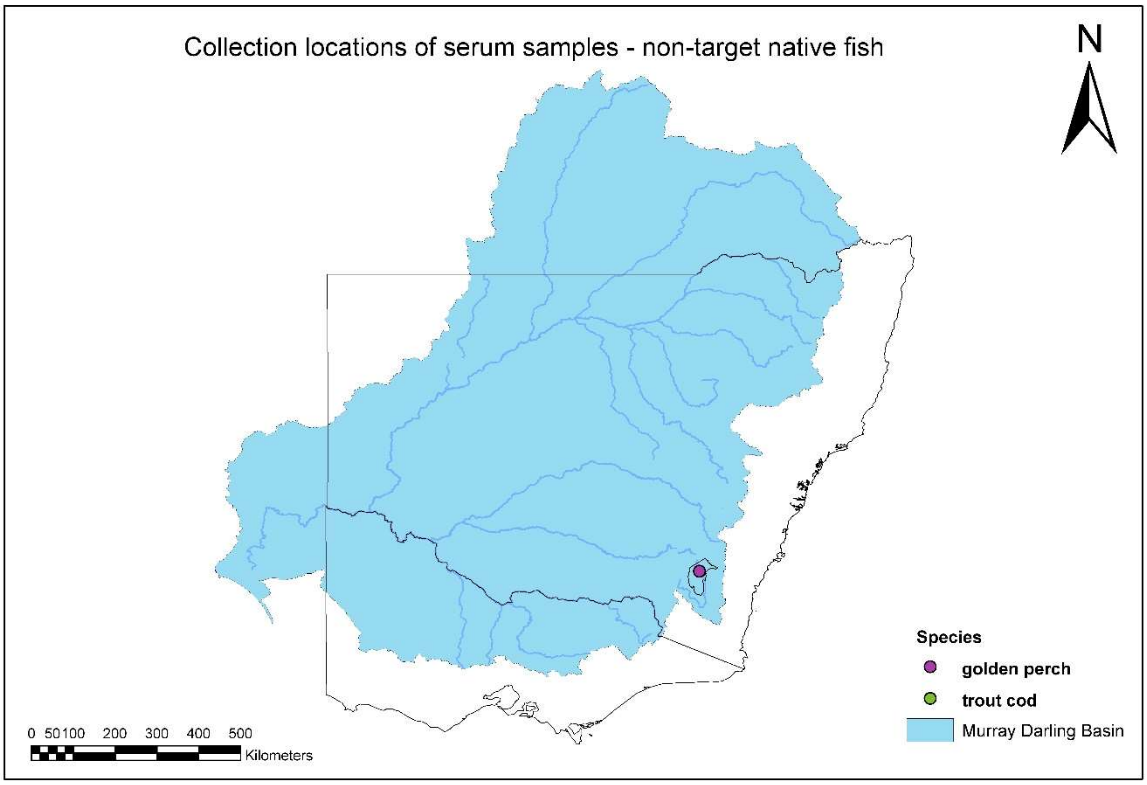

Supplementary Figure S6. Distribution of tissue and serum samples collected from other native fish species between July 2007 and June 2011. The locations for golden perch and trout cod were coincidental.
